# Modelling glioma progression, mass effect and intracranial pressure in patient anatomy

**DOI:** 10.1098/rsif.2021.0922

**Published:** 2022-03-23

**Authors:** Jana Lipková, Bjoern Menze, Benedikt Wiestler, Petros Koumoutsakos, John S. Lowengrub

**Affiliations:** ^1^ Department of Pathology, Brigham and Women’s Hospital, Harvard Medical School, Boston, MA, USA; ^2^ Dana-Farber Cancer Institute, Boston, MA, USA; ^3^ Broad Institute of Harvard and MIT, Cambridge, MA, USA; ^4^ Department of Informatics, Technical University of Munich, Munich, Germany; ^5^ Department of Quantitative Biomedicine, University of Zürich, Zürich, Switzerland; ^6^ Department of Neuroradiology, Klinikum Rechts der Isar, Technical University of Munich, Munich, Germany; ^7^ Computational Science and Engineering Lab, ETH Zürich, Zürich, Switzerland; ^8^ School of Engineering and Applied Sciences, Harvard University, Cambridge, MA 02138, USA; ^9^ Department of Mathematics, University of California, Irvine, CA, USA; ^10^ Department of Biomedical Engineering, University of California, Irvine, CA, USA; ^11^ Center for Complex Biological Systems, Chao Family Comprehensive Cancer Center, University of California, Irvine, CA, USA

**Keywords:** glioma, patient-specific modelling, tumour mass effect, brain deformation, intracranial pressure

## Abstract

Increased intracranial pressure is the source of most critical symptoms in patients with glioma, and often the main cause of death. Clinical interventions could benefit from non-invasive estimates of the pressure distribution in the patient's parenchyma provided by computational models. However, existing glioma models do not simulate the pressure distribution and they rely on a large number of model parameters, which complicates their calibration from available patient data. Here we present a novel model for glioma growth, pressure distribution and corresponding brain deformation. The distinct feature of our approach is that the pressure is directly derived from tumour dynamics and patient-specific anatomy, providing non-invasive insights into the patient's state. The model predictions allow estimation of critical conditions such as intracranial hypertension, brain midline shift or neurological and cognitive impairments. A diffuse-domain formalism is employed to allow for efficient numerical implementation of the model in the patient-specific brain anatomy. The model is tested on synthetic and clinical cases. To facilitate clinical deployment, a high-performance computing implementation of the model has been publicly released.

## 1. Introduction

Glioma is the most common type of primary brain tumour. Gliomas are divided into low- and high-grade tumours. High-grade gliomas (HGGs) are characterized by fast progression and poor prognosis with a median survival between 1 and 5 years, depending on the tumour subtype [1–3]. Low-grade gliomas (LGGs) progress at a slower pace and have a better prognosis; however, most LGGs eventually progress to HGGs [4]. In contrast to other primary malignancies, glioma manifests not only as an oncological but also as a neurological disease. The most common symptoms include: drowsiness (87%), progressive neurological (51%) and cognitive (33%) deficits, seizures (45%), incontinence (40%) and headaches (33%) [5–7]. The majority of the symptoms stem from increased intracranial pressure (ICP), which is also the main cause of death [8,9]. The corresponding mechanical forces further affect the tumour micro-environment and treatment efficacy [10]. The standard-of-care treatment of gliomas follows the Stupp protocol [1], which consists of combined surgery and chemo- and radiotherapy. Despite extensive efforts, gliomas are still incurable. Tumour management is complicated not only by the delicate nature of the brain tissue but also by the infiltrative growth of gliomas. In contrast to most cancers, gliomas do not form solid tumours with well-defined boundaries; instead, they infiltrate surrounding brain tissue even beyond the tumour borders visible on medical scans. The inability of current imaging methods to detect the whole tumour extent and pressure distribution contribute to the overall treatment complexity.

Computational tumour models can provide valuable insights into the patient's state and forecast the disease progression to assist clinical interventions. Most glioma models are based on the Fisher–Kolmogorov equation, which describes tumour proliferation and infiltration into the surrounding tissue [11–24]. Such models have been used to simulate spatio-temporal disease progression, response to treatment or transition from LGGs to HGGs [14,23,25–30]. The models can be further calibrated to patient-specific conditions to provide estimates about tumour infiltration pathways beyond the lesion outlines visible on medical scans [17,24,31–38]. Such estimates were, for instance, used to design personalized radiotherapy plans to spare healthy tissue and reduce radiation toxicity [31] or to predict response to treatment [35]. However, since these models do not account for the mechanical interactions between the growing tumour and surrounding brain tissue—the so-called tumour mass effect—their validity is limited to a short time scale before the tumour mass effect becomes significant. The clinical potential of glioma models can thus be significantly increased by accounting for the tumour mass effect and tumour-induced pressure in the patient's brain.

Although there is great interest in modelling brain biomechanical processes and deformations, not all existing models are suitable for glioma progression. For instance, a large body of models focus on modelling intraoperative brain deformations [39–43] or changes caused by traumatic brain injuries [44–46]. These models, however, consider biomechanical deformations caused by short-term forces acting from outside the skull, such as surgical loads, craniotomy-induced brain shift or impact head injury. Early models describing tumour-induced brain deformations originate from works by Mohamed *et al.* [47] and Hogea *et al.* [48], where brain tissue is modelled as an elastic material and tumour mechanical forces are approximated by a constant pressure acting on the tumour boundary. These models were designed for the construction of statistical brain atlases bearing tumours, and thus they do not account for tumour proliferation or infiltration. This issue was overcome by Clatz *et al.* [49], who combined the mechanical model with the Fisher–Kolmogorov equation describing tumour progression. The model was further updated by Abler *et al.* [50], who included additional linear coupling between tumour cell density and the growth-induced strain. In these models [49,50], however, the tumour dynamics is decoupled from the tissue deformation equation, which can lead to discrepancies, especially in the case of large deformations. A further improvement came from work by Hogea *at al.* [51,52], who used a nonlinear reaction–advection–diffusion equation to couple the tumour growth with brain tissue deformation, enabling simulations of large brain deformations. The model has been used in several simulation studies as well as medical image-processing tasks [33,53–56]. Recently, the model has been extended to account for different tumour components, such as proliferating, invasive and necrotic tumour cells, along with tumour-induced brain oedema, resulting in realistically appearing simulated tumours [57].

Despite considerable theoretical contributions, these models suffer from two main limitations. First, none of the previous models simulates the pressure distribution in the patient's brain. The existing models either do not consider the pressure term at all or the pressure is modelled as a constant increment, uniform in the whole brain, regardless of the tumour size or location. As a consequence, these models cannot provide any estimates of the pressure distribution or pressure-related symptoms, such as intracranial hypertension. Second, the models rely on a large number of parameters, which complicates their calibration to patient-specific conditions. Many parameters can be rendered patient- or disease-specific; however, inferring their values from the limited amount of non-invasive patient data is a challenging task. For instance, although medical scans reveal compression of brain tissues, this information might not be sufficient to identify the mechanical properties of individual tissue constituents as described by Young's modulus, Poisson’s ratio and other model parameters. Computational simulations thus usually rely on parameter values obtained from *in vivo* animal experiments or post-mortem studies. However, several studies have demonstrated large intra-species variations as well as rapid changes in brain tissue properties even a few minutes after death [58]. This in turn leads to a large variation in parameter values used across the literature [47,49,51], which further affects the ability of the computational models to provide patient-specific predictions.

To address these limitations, herein, we propose a novel glioma model that couples tumour dynamics, brain tissue deformation and corresponding ICP changes in the patient-specific anatomy. The model assumes that the growing tumour exerts pressure on the surrounding brain tissue, which is treated as a viscoelastic material. The brain tissues partially relax the pressure, depending on the tissue-specific properties, while the remaining pressure results in the deformation force. ICP is directly derived from the growing tumour, and it is constrained by patient-specific anatomy, enabling estimation of the pressure distribution in the whole patient parenchyma. The model predictions can be used to assess neurological and cognitive impairments caused by increased pressure in the patient-specific brain centres as well as predict onset of critical conditions such as intracranial hypertension (table 1) or brain midline shift [9,69]—a condition in which the brain moves towards one side of parenchyma, where a displacement above 5 mm usually requires immediate surgical intervention [70,71]. The estimated ICP distribution can help identify the regions with the highest pressure accumulation, which holds the potential to assist surgical interventions aiming to release the ICP.

**Table 1.** Relations between ICP values and corresponding pathological conditions [59–64]. While there are rare cases [65,66] with survival reported in patients with ICP >50 (mmHg), increased ICP is consistently associated with high mortality [62,67,68].

ICP (mmHg)	ICP (Pa)	condition
7–15	933–1999	normal
20–25	2666–3333	onset of hypertension
25–40	3333–5332	hypertension
40–50	5332–6666	loss of consciousness
>50	>6666	brain infraction and brain death

To facilitate model calibration for patient-specific predictions, the model uses a small number of parameters, as listed in table 2. Alongside the proposed model we present an efficient numerical formulation to facilitate the model implementation. The model is solved directly in the patient’s medical scans using the finite-difference (FD) method. In comparison, previous works used mainly finite-element methods since the FD approach often results in a large system of linear equations with a non-symmetric matrix, which complicates the numerical implementation [51]. The symmetry of the system is broken by the boundary conditions applied to the complex brain anatomy. To overcome this issue, we deploy the diffuse domain method (DDM) [72], which significantly reduces the numerical and computational complexity of the system. To further ease the model deployment, a high-performance model implementation is publicly released.^1^

**Table 2.** An overview of the model parameters and their units. The parameters *T* (day), *L* (cm) and *M* (g) denote the characteristic time, length and mass, respectively. The growing tumour u proliferates at rate *ρ* and infiltrates the surrounding brain tissue with tissue-specific infiltration rate D. As the tumour grows it exerts pressure  p on the surrounding brain tissue. The hydraulic conductivity M describes the ease with which the pressure passes through the brain tissues. Individual brain tissues partially relax the pressure, depending on the tissue-specific relaxation properties given by κ, while the resulting pressure leads to tissue translation and compression described by the deformation rate $\vec{v}$.

variable	units	description
u	*M*/*L*^3^	tumour cell density
ω	*M*/*L*^3^	brain tissue cell density
D	*L*^2^/*T*	tumour infiltration rate
*ρ*	1/*T*	tumour proliferation rate
p	*M*/*LT*^2^	tumour-induced pressure
κ	*T*/*L*^2^	pressure relaxation
$\vec{v}$	*L*/*T*	deformation rate
M	*L*^3^/*TM*	hydraulic conductivity

The rest of the paper is structured as follows: the proposed model is introduced in §2, followed by a description of the numerical implementation and the DDM formalism. The results are presented in §3, where the model is applied to synthetic and clinical cases. The relationship between ICP elevation and neurological symptoms is explored. The conclusion is presented in §4.

## 2. Methods

### 2.1. Biomechanical model

This section describes the proposed biomechanical model for glioma progression, mass effect and ICP dynamics in the patient-specific brain anatomy. The physics of the underlying process can be summarized as follows. The tumour proliferates and infiltrates the surrounding brain tissue, which consists of white matter, grey matter and cerebrospinal fluid (CSF). The brain tissue is assumed to consist of viscoelastic materials, where distinct tissue constituents are characterized by different mechanical properties. The growing tumour exerts pressure on the surrounding brain tissue. The pressure is partially relaxed by the brain tissue, depending on the tissue-specific mechanical properties, while the remaining pressure results in tissue deformation and compression. This mechanism entails three coupled processes: (i) tumour dynamics, (ii) pressure dynamics, and (iii) tissue dynamics; these represent the three main components of the model described in the following subsections.

**Figure 1.**
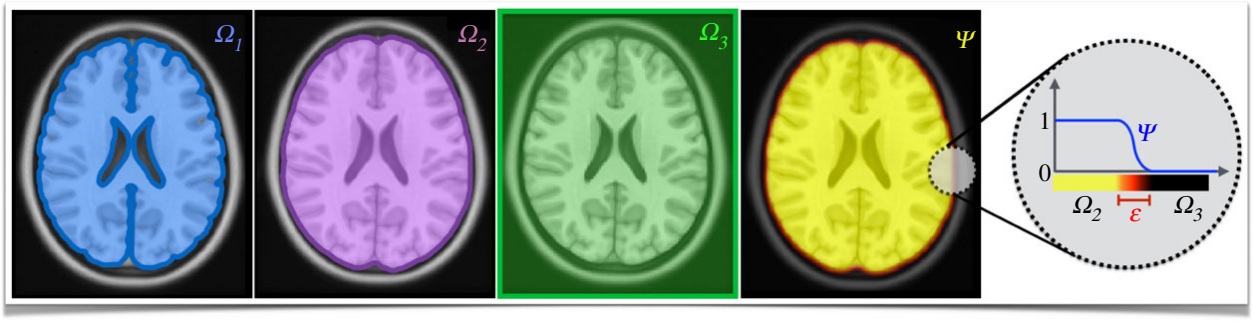
An overview of the simulation domains and the phase-field function. Three distinct simulation domains $\Omega_{1}\subset \Omega_{2}\subset \Omega_{3}\in R^{3}$ are considered to capture different processes. It is assumed that the tumour infiltrates only white and grey matter, which together constitute the domain *Ω*_1_ shown in blue. The tumour mass effect and pressure affect the whole-brain parenchyma; this is captured by domain *Ω*_2_, displayed in purple. A regular domain used for DDM formulation, *Ω*_3_, is portrayed in green. The last subplot shows the phase-field function ψ used to capture the relation between domains *Ω*_2_ and *Ω*_3_.

To facilitate the model description, the rest of this paragraph outlines the notation and assumptions. The model is solved in the patient-specific brain anatomy reconstructed from magnetic resonance imaging (MRI) scans, where each image voxel corresponds to one simulation grid point. The letter *i* ∈ {1, …, *N*} denotes an index across all *N* voxels of the MRI scan. It is assumed that the tumour cells infiltrate only white and grey matter, whereas the pressure affects all the tissues in the brain parenchyma. According to this assumption, we consider three distinct simulation domains: $\Omega_{1}(t)\in R^{3}$, a domain consisting of white and grey matter only; $\Omega_{2}\in R^{3}$, a domain including white matter, grey matter and CSF (i.e. the whole brain parenchyma); $\Omega_{3}\in R^{3}$, a regular domain containing the brain parenchyma and skull (e.g. whole brain scan). Figure 1 shows the three simulation domains, and it is assumed that *Ω*_1_(*t*) ⊂ *Ω*_2_ ⊂ *Ω*_3_. We note that the border of the domain *Ω*_1_(*t*) changes over time *t* as a result of the tumour-induced changes, such as tissue displacement. On the other hand, the borders of the domains *Ω*_2_ and *Ω*_3_ are constant over time, since *Ω*_2_ is constrained by a rigid skull and *Ω*_3_ is defined as a fixed domain. To distinguish the different scopes of model variables, the following labelling is used: the field variables defined at every voxel, such as tumour cell density, are marked by bold letters, e.g. x; the vectors of field variables, such as deformation rate, are denoted by bold letters with an arrow, e.g. $\vec{x}$; scalar variables, such as the proliferation rate, are marked by non-bold letters, e.g. *x*. An overview of the model parameters is provided in table 2.

#### 2.1.1. Tumour dynamics

The glioma dynamic consists of tumour proliferation and infiltration into the surrounding brain tissue. Let *u*_*i*_(*t*) ∈ [0, 1] be a normalized tumour cell density at time *t* and voxel *i* ∈ {1, …, *N*} at location (*i*_*x*_, *i*_*y*_, *i*_*z*_) ∈ *Ω*_1_(*t*). The dynamics of the tumour cell density $u\;:={\left\{u_{i}(t)\right\}}_{i=1}^{N}$ in the brain tissue *Ω*_1_(*t*) is modelled as:

2.1 $$\frac{\partial u}{\partial t}=\nabla \cdot (D\;\nabla u)+\rho u(1-u)-\vec{v}\cdot \nabla u\;\text{in}\;\Omega_{1}(t),$$

2.2 $$\nabla u\cdot \vec{n}=0\;\text{in}\;\partial \Omega_{1}(t),$$

2.3 $$u(t=0)=u_{0}\;\text{in}\;\Omega_{1}(t=0).$$

The first term on the right-hand side of equation (2.1) describes tumour infiltration into the surrounding brain tissue, where $D={\left\{D_{i}(t)\;I\right\}}_{i=1}^{N}$ is a tissue-dependent tensor, I is a 3 × 3 identity matrix and

2.4 $$D_{i}(t)=\left\{ \begin{array}{cc} p_{w_{i}}(t)\;D_{w}+p_{g_{i}}(t)\;D_{g}\; & \text{for }\;i\in \Omega_{1}(t),\\ 0\; & \text{ for }\;i\notin \Omega_{1}(t),\; \end{array}\right.$$

where $p_{w_{i}}(t)$ and $p_{g_{i}}(t)$ denote the percentage of the white and grey matter at voxel *i* at time *t*. The constants *D*_*w*_ and *D*_*g*_ describe the tumour infiltration rate in white and grey matter, respectively. Since the tumour cells infiltrate the white matter faster than the grey matter, it is assumed that $D_{w}=10D_{g}\;({\mathrm{cm}}^{2}\;{\mathrm{day}}^{-1})$ [24,73]. Let us note that the tissue percentages $p_{w_{i}}(t)$ and $p_{g_{i}}(t)$ change over time owing to the tumour-induced tissue displacement. The second term on the right-hand side of equation (2.1) describes self-limiting tumour proliferation, where *ρ* (1/day) is the proliferation rate. As the tumour grows, it exerts pressure on the surrounding tissue, which is deformed and displaced in response. The brain tissue displacement also causes displacement of the tumour cells infiltrated inside that tissue. This is modelled by the last term on the right-hand side of equation (2.1), where $\vec{v}\;:={\left\{(v_{x_{i}}(t),v_{y_{i}}(t),v_{z_{i}}(t)\;)\right\}}_{i=1}^{N}$ is the rate of displacement. The skull and CSF (including ventricles) are not infiltrated by the tumour and act as a domain boundary with an imposed no-flux boundary condition given by equation (2.2), where $\vec{n}$ is the unit outward normal to ∂*Ω*_1_(*t*). The tumour is initialized as a point source at voxel *i*_*ic*_ ∈ *Ω*_1_(*t* = 0) and its growth is modelled from the initial time *t* = 0 until the final time *t* = *T* (day).

#### 2.1.2. Pressure dynamics

The pressure dynamics are determined by the growing tumour pressing on the surrounding brain tissue and the mechanical properties of the individual tissue constituents. The brain tissue acts as a viscoelastic material that partially relaxes the pressure from the tumour. The pressure relaxation is proportional to the tissue-specific mechanical properties—the softer the material the more pressure it relaxes. The remaining pressure results in tissue deformation. The deformation rate can be computed from the pressure gradient using Darcy’s law. Let $\vec{v}$ denote the deformation rate. For the pressure term, we will assume that  p(t=0) is the patient’s normal pressure before the onset of the tumour and $p^{\prime }(t)$ is the patient’s pressure with the tumour at time *t*. The tumour-induced pressure  p(t) is then given as $\;p(t)={\;p}^{\prime }(t)-\;p(t=0)$. Since the exact values of the patient’s normal pressure are not known, we will assume  p(t=0)=0 and use the following equations to simulate the dynamics of the tumour-induced pressure  p(t) in the domain *Ω*_2_:

2.5 $$\vec{v}=-M\cdot \nabla \;p\;\text{in}\;\Omega_{2},$$

2.6 $$\nabla \cdot \vec{v}=\rho \;u(1-u)-\varkappa \;\;p\;\text{in}\;\Omega_{2},$$

2.7 $$\vec{v}=0\;\text{on}\;\partial \Omega_{2}.$$

The parameter M, sometimes referred to as hydraulic conductivity, describes the ease with which the pressure passes through the tissue. The pressure relaxation is described by the parameter $\varkappa \;:={\left\{\varkappa_{i}(t)\right\}}_{i=1}^{N}$, where

2.8 $$\varkappa_{i}(t)=\left\{ \begin{array}{cc} p_{w_{i}}(t)\;\varkappa_{w}+p_{g_{i}}(t)\;\varkappa_{g}+p_{c_{i}}(t)\;\varkappa_{c}\; & \text{for }\;i\in \Omega_{2},\;\\ 0\; & \text{for }\;i\notin \Omega_{2}.\; \end{array}\right.$$

The terms $p_{w_{i}}(t),p_{g_{i}}(t),p_{c_{i}}(t)$ denote the percentage of white matter, grey matter and CSF at voxel *i* at time *t*, while $\varkappa_{w},\varkappa_{g}$ and $\varkappa_{c}$ denote the relaxation rate of the corresponding tissues. The softer the material is, the more pressure it relaxes; thus, it is assumed that $\varkappa_{c}>\varkappa_{w}>\varkappa_{g}$. For simplicity, we assume constant hydraulic conductivity $M=8.53\times 10^{-9}\;({\mathrm{cm}}^{2}\cdot {\mathrm{mmHg}}^{-1}\cdot s^{-1})$, as reported in [74]. This means that the tumour exerts the same pressure on all surrounding tissues, but distinct tissues respond differently depending on their mechanical properties. By substituting equation (2.5) into both equations (2.6) and (2.7) we obtain a Helmholtz-like equation, which allows the computation of the pressure field as

2.9 $$-\nabla \cdot (M\cdot \nabla \;p)=\rho \;u(1-u)-\varkappa \;p\;\text{in }\Omega_{2},$$

2.10 $$\nabla \;p\cdot \vec{n}=0\;\text{on }\;\partial \Omega_{2}.$$

The pressure  p obtained by solving equations (2.9) and (2.10) is used to compute the deformation rate $\vec{v}$ from (2.5), which is consequently used to model the tissue deformation.

#### 2.1.3. Tissue dynamics

The growing tumour compresses and displaces the surrounding brain tissue. Let $\omega_{w_{i}}(t),\omega_{g_{i}}(t)$ and $\omega_{c_{i}}(t)$ denote the cell density of white matter, grey matter and CSF, respectively, at voxel *i* ∈ *Ω*_2_ at time *t*. The dynamics of each tissue constituent $\omega_{s}\;:={\left\{\omega_{s_{i}}(t)\right\}}_{i=1}^{N}$, *s* ∈ {*w*, *g*, *c*} is modelled by the following advection–convection equation:

2.11 $$\frac{\partial \omega_{s}}{\partial t}=-\vec{v}\cdot \nabla \omega_{s}-\omega_{s}\nabla \cdot \vec{v}\;\text{in}\;\Omega_{2},$$

2.12 $$\nabla \omega_{s}\cdot \vec{n}=0\;\text{on}\;\partial \Omega_{2},$$

2.13 $$\omega_{s}(t=0)=\omega_{s_{0}}\;\text{in}\;\Omega_{2}.$$

The cell density of each tissue component in equation (2.13) is initialized from the tissue segmentation, which can be computed with the provided open-source software.^2^ The advection term describes tissue displacement, while the convection term models tissue compression. After each simulation time step, the percentage of each tissue component must be recomputed accordingly, i.e. for $\forall i\in \Omega_{2}$ and *s* ∈ {*w*, *g*, *c*}

2.14 $$p_{s_{i}}(t)=\frac{\omega_{s_{i}}(t)}{\omega_{w_{i}}(t)+\omega_{g_{i}}(t)+\omega_{c_{i}}(t)}.$$

This ensures that the tissue percentage at each voxel is compatible with the tissue density maps and that $p_{w_{i}}(t)+p_{g_{i}}(t)+p_{c_{i}}(t)=1$ for $\forall i\in \Omega_{2}$.

Let us note that in equation (2.1) the tumour is only subject to displacement, not compression. The reason for this assumption is that gliomas, in contrast to other solid tumours, infiltrate the surrounding tissue, and thus the pressure acting from the tissue on the tumour is minimal. However, if tumour compression is of interest, this can be achieved by including the convection term (i.e. $-u\nabla \cdot \vec{v}$) on the right-hand side of equation (2.1).

#### 2.1.4. Further considerations

The model can be further updated to account for the anisotropic tumour cell migration along the fibres in the white matter. This can be achieved by replacing the identity matrix I by a tensor constructed based on the water diffusion tensor obtained through diffusion tensor imaging (DTI), as done in previous works [49,75–78]. Patient-specific DTI scans have the potential to inform the speed and direction of the tumour infiltration in the white matter. There are, however, two reasons why DTI-based infiltration is not considered in our model. First, since DTI measures water diffusion, the DTI signal is corrupted in the oedematous regions owing to increased water content, which can have an adverse effect on the simulated infiltration in the tumour region and its proximity. Second, it is not clear how the orientation of the tissue fibres is affected during tissue compression and deformation caused by the growing tumour. Therefore, in this paper, the tumour infiltration is only constrained by anatomical structures, whereas the deployment of DTI in the presence of the tumour mass effect might require further studies.

Additional model updates can be achieved by including a damping effect in the diffusion term informed by von Mises stress, as presented in [35,79]. This approach accounts for the inhibition in tumour expansion caused by local tissue stress, as observed experimentally for solid tumours [80]. However, in the case of diffusive glioblastomas [81,82] and some other cancers [83,84], experiments suggest increased tumour invasiveness in response to elevated local pressure and compression. More studies are thus required to fully understand the tumour behaviour in relation to its micro-environment and computational models such as [35,79] can assist such studies. In this paper, we consider only tissue-dependent tumour infiltration to keep the number of model parameters minimal.

### 2.2. Numerical formulation

This section presents how the DDM can be deployed to reduce the computational intensity of the proposed model. Details of the numerical implementation are discussed afterwards.

#### 2.2.1. Diffuse domain formulation

The computational cost of the proposed model is largely determined by the numerical solution of the Helmholtz equations (2.9) and (2.10). Its numerical discretization with the FD method leads to a system of linear equations with a non-symmetric matrix. The non-symmetry is due to the complex brain anatomy and the imposed no-flux boundary condition (equation (2.10)). In turn, many efficient numerical methods such as Jacobi, Gauss–Seidel or conjugate gradient methods cannot be used since they all require symmetric matrices. However, this issue can be efficiently overcome by the DDM [72]. The DDM offers a way to replace a system of equations defined on a complex domain by a new set of equations defined on an arbitrary regular domain with desired boundary conditions. In our case, the Helmholtz equation defined at *Ω*_2_ can be replaced by a new set of equations defined on regular domain *Ω*_3_ with a zero Dirichlet boundary condition on ∂*Ω*_3_, resulting in a system of linear equations with a symmetric matrix. The DDM method uses a so-called phase-field function $\psi \;:={\left\{\psi_{i}\right\}}_{i=1}^{N}\in \Omega_{3}$ to capture the relationship between the domains *Ω*_2_ and *Ω*_3_. The phase-field function ψ is a smoothed version of the characteristic function $\chi_{{|}_{\Omega_{2}}}$ of the domain *Ω*_2_, i.e. *ψ*_*i*_ = 1 for *i* ∈ *Ω*_2_, *ψ*_*i*_ = 0 for $i\in \Omega_{3}\setminus \Omega_{2}$, and *ψ* varies smoothly at the interface between the domains *Ω*_2_ and *Ω*_3_, such that *ψ*(*i*) = 0.5 for *i* ∈ ∂*Ω*_2_, as shown in figure 1. Using the DDM, the Helmholtz equation given by equations (2.9) and (2.10) is reformulated as

2.15 $$-\nabla \cdot (M\cdot \psi \nabla \;p)=\psi \rho \;u(1-u)-\psi \varkappa \;p\;\text{in }\;\Omega_{3},$$

2.16 $$\;p=0\;\text{on }\;\partial \Omega_{3}.$$

The FD discretization of the above equations leads to a system of linear equations with a symmetric matrix, thus reducing significantly the numerical complexity of the problem. The phase-field function ψ, like *Ω*_2_, does not change over time since it is constrained by a rigid skull. The pressure computed by the above equations in domain *Ω*_3_ can be easily mapped to domain *Ω*_2_ (i.e. brain parenchyma) by multiplication with the characteristic function $\chi_{{|}_{\Omega_{2}}}$. The phase-field function can be obtained by solving the Cahn–Hilliard equation [85,86],

2.17 $$\frac{\partial \psi }{\partial t}=\nabla \cdot (A(\psi )\cdot \nabla (g^{\prime }(\psi )-\varepsilon^{2}\Delta \psi ))\in \Omega_{3},$$

2.18 $$\psi (t=0)=\left\{ \begin{array}{cc} 1\; & \text{on }\;\Omega_{2},\;\\ 0\; & \text{on}\;\Omega_{3}\setminus \Omega_{2},\; \end{array}\right.$$

where $g(\psi )=\frac{1}{4}\psi^{2}{\left(1-\psi \right)}^{2}$ is a double-well potential and the term A(ψ) controls the behaviour of the phase-field function at the interface, while *ɛ* is the prescribed interface thickness. Taking $A(\psi )=\sqrt{4g(\psi )}$ limits interface displacement under the Cahn–Hilliard dynamics. The Cahn–Hilliard equation is solved in time until the phase-field function reaches a smooth interface with the prescribed thickness. The desired width of the interface depends on the application; in this case, the interface width of 3 voxels (i.e. 3 mm) is considered.

**Algorithm 1.** Implementation of the tumour-induced brain deformation model.

1. *Initialise*: t=0, $u(t=0)=u_{0}$, $\omega_{s}(t=0)=\omega_{s_{0}}$ for s∈{w, g, c}
2. Compute tissue percentage by equation (2.14)
3. **while** (t≤T) **do**
4. Compute time step $\tau =\min(\tau_{1},\;\tau_{2})$, where $\tau_{1}$ and $\tau_{2}$ are time steps constrained by the numerical stability of equations (2.1) and (2.11)
5. Compute the **pressure** p(t) by solving equations (2.15) and (2.16)
6. Compute the **deformation field** $\vec{v}(t)$ by solving equations (2.5) and (2.7)
7. Update the **tumour** state by solving equations (2.1) and (2.2)
8. Update the **brain tissue** by solving equations (2.11) and (2.12)
9. Recompute the **tissue percentage** by equation (2.14)
10. t=t+τ
11. **end while**

To simplify the process and to avoid re-computation of the phase-field function for each new patient, affine image registration can be used instead. More specifically, we provide the brain MRI atlas with the binary brain mask (i.e. $\chi_{|\Omega_{2}}$) and the corresponding phase-field function computed by equation (2.17).^1^ For each new patient, the patient-specific phase-field function can be obtained by computing an affine registration, F, that maps the atlas brain mask to the patient's brain mask. The resulting registration F is then used to map the pre-computed atlas phase-field function to the patient's brain, leading to the patient-specific phase-field function. To ease the process, an automated image-registration software as well as brain atlas are provided.^2,^^3^

We remark that the DDM approach is used only for the Helmholtz equation. In principle, the whole model could be expressed in the DDM formalism. However, if the simulation domain contains very fine anatomical structures, it might not be possible for the phase-field function to have a smooth interface and preserve the structures at the same time. For instance, the separation between frontal brain lobes often consists of only 1–2 voxels in some regions, and thus a phase-field function with ɛ of even 1 voxel could distort the separation between the hemispheres, creating artificial pathways for tumour infiltration.

#### 2.2.2. Implementation

A numerical implementation of the proposed model is outlined in algorithm 1, while the software is publicly available.^1^ The software uses the FD method for the space discretization together with forward Euler time integration. The Helmholtz equation, given by equations (2.15) and (2.16), is solved with a multigrid preconditioned conjugate gradient method [87] using the *hypre* library [88]. The advection–convection equation (2.11) is solved with the fifth-order weighted essentially non-oscillatory (WENO5) scheme [89]. The advantage of the WENO5 scheme is its capability to achieve highly accurate solutions in smooth regions while maintaining stable, non-oscillatory transitions in regions with sharp discontinuities [90], which appear for instance at interfaces between the CSF and brain tissue. A high-performance implementation is achieved through a hybrid OpenMP and MPI parallelization. For the software and tutorial, please refer to our repository.^1^

## 3. Results

The proposed model is tested on synthetic and clinical cases. The synthetic case is used to illustrate the model's capability of simulating tumour progression and the corresponding mass effect. The relation between ICP elevation and neurological symptoms is explored. The model is then applied to clinical cases of patients with HGG and LGG lesions. The HGG cases exhibit significant brain deformations; however, owing to the aggressive nature of the disease only scans from a single time point prior to treatment are available. These cases are used to demonstrate the model’s capability of reproducing realistic and large brain deformations. Afterwards, data showing tumour progression from LGG to HGG are used to assess the potential of the model to capture disease progression over time. The synthetic case uses healthy brain anatomy obtained from the SRI24 Atlas [91], while the patient cases are obtained from the Brain Tumour Segmentation (BraTS) challenge [92,93].

### 3.1. Synthetic case

For the synthetic case, brain anatomy from a healthy subject provided by the SRI24 Atlas is used to simulate the disease progression over time. The brain tissue segmentation is provided together with the atlas. The tumour is initialized as a point source in the right frontal lobe and its progression is simulated over a period of 600 days using the following model parameters: *D*_*w*_ = 1.3 × 10^−3^ (cm^2^/day), *D*_*g*_ = 1.3 × 10^−4^ (cm^2^/day), *ρ* = 1.2 × 10^−2^ (1/day), $\varkappa_{g}=2.0\times 10^{-3}\;(\mathrm{day}/{\mathrm{cm}}^{2})$, $\varkappa_{w}=2.0\times 10^{-2}\;(\mathrm{day}/{\mathrm{cm}}^{2})$, $\varkappa_{c}=2.0\times 10^{-1}\;(\mathrm{day}/{\mathrm{cm}}^{2})$ and $M=8.53\times 10^{-9}\;({\mathrm{cm}}^{2}\cdot {\mathrm{mmHg}}^{-1}\cdot s^{-1})$. The values of parameters (*D*_*w*_, *D*_*g*_, *ρ*) were taken from [31,94] and M from [74], while values of $\varkappa_{w},\varkappa_{g},\varkappa_{c}$ were chosen empirically.

**Figure 2.**
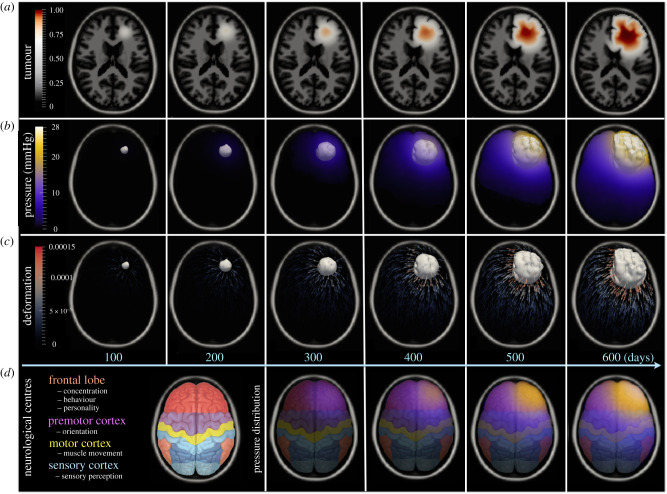
Simulation of disease progression over 600 days in the synthetic case. The subplot (*a*) shows a two-dimensional slice of the brain anatomy with the tumour, while the colour bar indicates the tumour cell density. Tumour progression results in compression of the right ventricle and brain midline displacement in the frontal lobe, the latter one being more visible in figure 3. The pressure distribution and deformation field, together with the tumour outline, shown as the white iso-surface given by *u* = 0.3, are depicted in three dimensions in the subplots (*b*) and (*c*), respectively. The pressure values correspond to the tumour-induced pressure increase over the subject's ICP before the onset of disease. The subplot (*d*) shows neurological brain centres mapped to the patient’s anatomy, followed by a visualization of the pressure distribution superimposed on the top of the specific neurological centres. For visualization purposes, the last row shows the pressure distribution from day 300 onwards.

**Figure 3.**
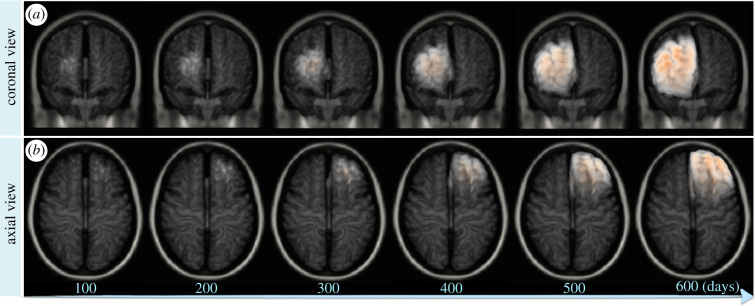
Visualization of the brain midline displacement over time in the synthetic case. The results correspond to the same subject and simulation depicted in figure 2. The three-dimensional visualizations, provided in (*a*) coronal and (*b*) axial view, show brain midline shift, which is one of the indicators of critical conditions. For visualization purposes, the CSF is not depicted.

Figures 2 and 3 show disease progression over time in two- and three-dimensional views. The mass effect of the growing tumour causes brain deformations, including ventricle compression and brain midline displacement, where the latter is more visible in the three-dimensional visualization shown in figure 3. The resulting tumour morphology has a complex pattern compatible with the brain anatomy. The ICP, shown in figure 2*b*, is directly derived from the growing tumour and is constrained by the patient-specific brain anatomy. The model identified the highest pressure accumulation in the frontal lobe, where the growing tumour compresses the brain tissue against the inner wall of the skull. On the other hand, the pressure in the ventricles is much lower, which is consistent with the capability of the CSF to relax the pressure [95]. The deformation field, shown in figure 2*c*, is most dominant at the interface between the tumour and the tissues, i.e. the region where the bulk tumour presses most on the surrounding tissue, while its effect decreases with increasing distance from the tumour. The model predictions can be used to assist clinical interventions, such as ICP management or surgery planning. The distribution of the ICP can be used to estimate the neurological symptoms caused by the increased pressure in the specific brain centres, as shown in figure 2*d*. For instance, in the presented case the pressure elevation in the frontal lobe, as seen in the initial stages of the disease, can affect the subject’s behaviour and concentration. As the pressure elevation progresses to the motor cortex, deterioration of motor functions can be expected, followed by impairment of perception caused by further disease progression to the sensory cortex in the parietal lobe. Interestingly, the identified ICP values are also comparable to the pathological values reported in table 1. The latter time points show intracranial hypertension, which is also compatible with the brain midline displacement (shown in figure 3), both representing critical conditions. For clinically relevant predictions, however, model calibration to the patient-specific conditions is needed. This example illustrates the potential of the model to simulate tumour progression, together with the brain deformations and ICP increase over time.

### 3.2. High-grade glioma cases

**Figure 4.**
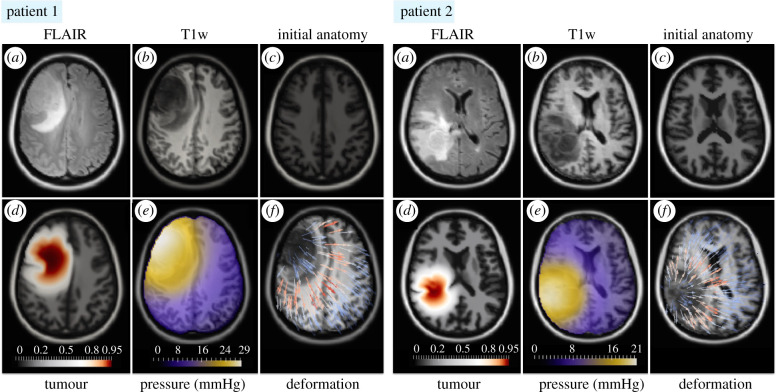
Simulation of disease progression in two patients with HGG. The subplots (*a*,*b*) show FLAIR and T1w MRI scans of each patient from the time of the tumour detection. Since the scans with patient brain anatomy before the onset of the disease are not available, affine registration of the brain atlas to the patients' preoperative MRI scans is used to approximate the tumour-free brain anatomy. The resulting tumour-free brain anatomy is shown in subplots (*c*) and serves as the initial condition for the simulation. The tumour is initialized as a point source in the centre of mass of the FLAIR-enhancing lesion and its progression is simulated over time until it reaches a mass comparable to the tumour visible on the patients' scans. Subplot (*d*) shows the simulated tumour and its effect on the surrounding brain tissue. The colour bar indicates the tumour cell density. The subplots (*e*,*f*) show the simulated pressure distribution and the deformation field overlaid on the patients' T1w MRI scans. The predicted brain deformations mimic the state of the disease visible in the patients' MRI scans. For each patient, the slice across the tumour centre of mass is shown.

To assess the ability of the model to produce realistic brain deformations, the model is used to reproduce the tumour mass effect observed in MRI scans of patients diagnosed with HGG. Figure 4*a*,*b* shows fluid-attenuated inversion recovery (FLAIR) and T1-weighted (T1w) MRI scans of two patients at the time of tumour detection. The FLAIR scan provides good contrast between healthy tissue and tumour (seen as bright enhancement), while the contrast between white and grey matter is less pronounced. On the other hand, the T1w MRI shows lower enhancement in the lesion but superior contrast between the distinct brain tissues. The FLAIR scan is therefore used to detect the tumour extent while the T1w MRI is deployed to estimate the brain anatomy.

To perform the simulation, we would like to initialize the tumour in the patient’s tumour-free non-deformed anatomy and model the disease progression over time, until it reaches the state visible on the patient’s scans. Since the scans with the patient’s healthy anatomy are usually not available, we approximate the initial deformation-free anatomy through image registration. More specifically, the T1w MRI scan from the SRI24 atlas [91] is mapped to the patient’s T1w scan using affine ANTs [96] registration.^3^ The approximated healthy anatomies of both patients are shown in figure 4*c*. This approach provides a reasonable approximation of the deformation-free anatomy in most cases. However, the affine registration might not be sufficient to capture atypical anatomical morphologies, such as enlarged ventricles, which might require manual correction. In the simulation, the tumour is initialized as a point source at the centre of mass of the FLAIR-enhancing lesion. The time of the tumour growth, from its onset to detection, is not known in practice. However, from the simulation perspective, one can obtain the same tumour morphology with different combinations of time-dependent parameters, such as *T* and speed of growth, i.e. (*D*_*w*_, *ρ*), as shown in [31,73]. Since the aim of these examples is to illustrate the potential of the model to generate realistic brain deformations, the same parameters as in the synthetic case are used; except the final time *T,* which is chosen manually by comparing the extent of the real and the predicted tumour.

The results of the model simulations for both cases are shown in figure 4*d*–*f*. The predicted tumour mass effect is in good qualitative agreement with the tumour-induced deformations observed in the patients’ MRI scans. In the case of patient 1, the model correctly predicts the midline displacement, while for patient 2 it accurately reproduces the compression of the ventricles. The simulated tumours have complex morphology similar to tumour patterns seen in the patients’ scans. Figure 4*e* shows the predicted ICP distribution overlaid on the patients’ T1w MRI scans. The estimated pressure distribution closely follows the morphology of the visible tumour, with the highest pressure accumulation mainly in the tumour core close to the skull. The high ICP values imply intracranial hypertension in both patients (table 1), which is a reasonable estimate given the large size of the lesions. The deformation field, shown in figure 4*f*, is most pronounced at the outlines of the visible tumour, i.e. the regions where the bulk tumour presses most on the surrounding tissue. Small deviations can be attributed to the discrepancies in the approximation of the initial tumour-free brain anatomy. For instance, the ventricles in the frontal lobe of patient 2 appear smaller in the approximated initial anatomy than in the patient’s scans. These examples demonstrate the feasibility of the model to reproduce a realistic tumour mass effect, even in the presence of large deformations.

### 3.3. Low-grade glioma case

**Figure 5.**
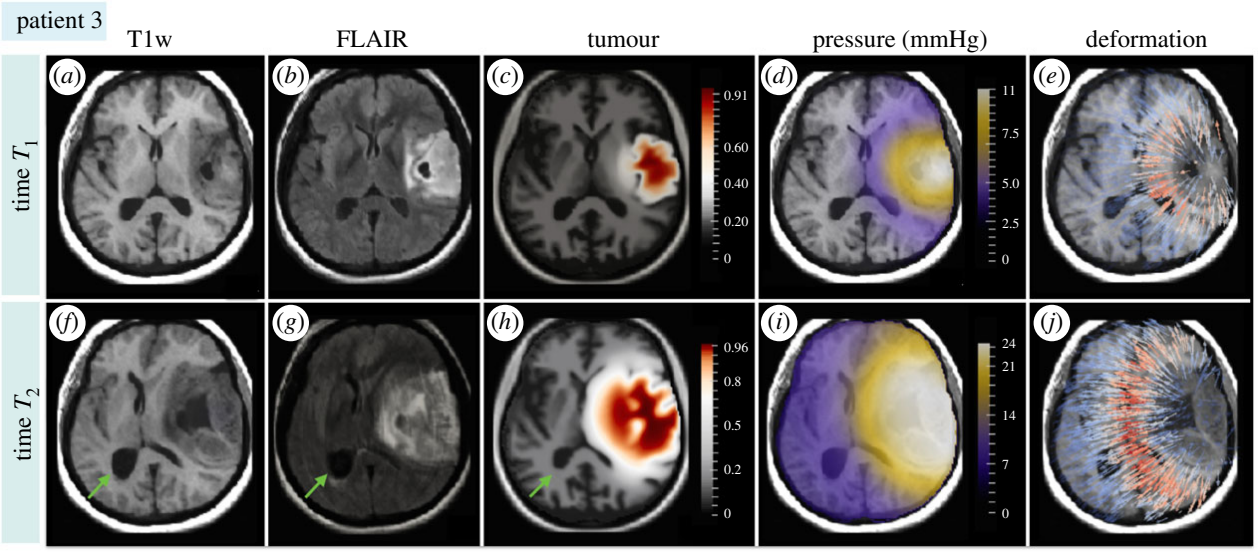
Simulation of disease progression over time. Subplots (*a*,*b*) and (*f*,*g*) show the patient’s T1w and FLAIR MRI scans acquired at two different time points, *T*_1_ (the first row) and *T*_2_ (the second row). The first time point shows LGG at the time of the tumour detection, while the second time point depicts the tumour progression to HGG. For the simulation, the tumour is initialized as a point source in the deformation-free anatomy, similarly to the previous cases, and its progression is simulated from the time of disease onset (i.e. *t* = 0 (days)) through time *t* = *T*_1_ to time *t* = *T*_2_. The specific values for parameters *T*_1_ and *T*_2_ are found manually by comparing the mass of the simulated and the patient's tumour visible on the scans. Results of the simulation at *T*_1_ = 200 (days) and *T*_2_ = 700 (days) are depicted in (*c*–*e*) and (*h*–*j*), respectively. Subplots (*c* and *h*) show the simulated tumour and its effect on the surrounding anatomy for each corresponding time point. The colour bars indicate the tumour cell density. The model correctly predicts a small mass effect at time *T*_1_, while at time *T*_2_ it correctly captures the compression of the right ventricle and the midline shift. However, the model did not capture the enlargement of the left ventricle at time *T*_2_ (marked by green arrows), which stems from the changes in CSF circulation. Subplots (*d*,*e*) and (*i*,*j*) show the predicted pressure distribution and the deformation field visualized on top of the patient’s T1w MRI scans from the corresponding time points. At time *T*_1_, the model predicts mild hypertension, which is compatible with low-grade tumours, while more advanced hypertension is estimated as the tumour progresses to an HGG at time *T*_2_. The deformation field is most prominent at the borders of the visible tumour, i.e. the region where the bulk tumour presses most on the surrounding tissue, indicating the deformations towards the brain midline. This example demonstrates that, except for the changes in the CSF circulation, the model can capture disease progression over time.

We test the feasibility of the model to capture the disease progression over time in a patient case with MRI scans from two different time points. Figure 5*a*,*b*,*f*,*g* shows the patient's scans, where the first time point *T*_1_ corresponds to an LGG lesion from the time of disease detection, while the second time point *T*_2_ shows the tumour transition to HGG. We note that the glioma scans, showing tumour progression over multiple time points, are relatively rare since patients usually undergo treatment or tumour resection. Since in this case data from two time points are available, the scan from the first time point *T*_1_ could be used to initialize the simulation, both the tumour cell density and the brain anatomy, and the model would simulate the disease progression to the second time point *T*_2_. This approach, however, poses a few challenges. First, the patient's brain anatomy below the visible tumour is not known. Second, since the MRI scan captures only the tumour morphology, the tumour cell density and the tumour infiltration beyond the visible tumour borders are unknown. Such uncertainties in the initial condition would significantly affect the model prediction. Moreover, since these scans come from a public database focused on tumour segmentation, the acquisition time of the scans is not provided. But even if the time span between the two time points were known, the disease most likely does not proceed at a constant pace owing to treatment. Since the aim of this example is to assess the ability of the model to capture tumour progression over time, rather than provide patient-specific predictions, a similar strategy as in the previous cases is applied. In particular, the tumour is initialized as a point source in the deformation-free anatomy and this time point is considered as the initial time *t* = 0 (days). The initial brain anatomy is obtained by mapping the brain atlas to the patient's T1w scan from the first time point, i.e. *T*_1_. The initial tumour location is chosen as the centre of mass of the tumour visible on the FLAIR scan from time *T*_1_. The tumour progression is simulated from the time of disease onset, i.e. *t* = 0 (days), through the time of the tumour detection at *t* = *T*_1_ (days) until the final time *t* = *T*_2_ (days) using the same parameter values as in the previous cases. The time points corresponding to the patient's scans were found manually by comparing the extent of the simulated and real tumour, and were estimated as *T*_1_ = 200 (days) and *T*_2_ = 700 (days). The additional benefit of this approach is that the model predictions can be evaluated at two different time points instead of one.

The results of the simulation at time points *T*_1_ and *T*_2_ are shown in figure 5. The model accurately predicts a small tumour mass effect at the first time point, which is consistent with the LGG visible on the patient’s first scan. The estimated pressure distribution and the deformation field follow the morphology of the actual tumour. The moderate elevation in the ICP implies the onset of intracranial hypertension (table 1). At the second time point, the model correctly captures the disease progression, including the compression of the right ventricle and the brain midline shift. The increased ICP indicates advanced intracranial hypertension, typical for HGG, with a high-pressure accumulation in the whole tumour core. However, the model did not capture the enlargement of the left ventricle in the second time point, marked by the green arrows in figure 5. Such ventricular enlargement happens in some patients owing to changes in CSF circulation, which can be caused either by the tumour itself or as a response to treatment [97]. Since the model does not account for the CSF circulation such deformations are not captured. This example shows that, except for ventricular enlargement, the model can provide realistic predictions about disease progression over time. Such predictions can serve as the worst-case scenario estimates about disease progression in the absence or insufficiency of treatment.

## 4. Conclusion

This paper proposes a novel model coupling glioma progression, tumour mass effect and ICP dynamics. Synthetic and clinical cases are used to illustrate the model’s capability to capture realistic disease progression and brain deformations and to estimate the ICP distributions. The main advantage of the proposed approach is that the ICP is directly derived from the tumour dynamics and the patient-specific anatomy. This allows for estimates of critical conditions such as hypertension, brain midline shift or neurological impairments caused by high-pressure accumulation in specific brain areas. The distribution of ICP has further potential to guide surgical interventions.

The model could also be used to guide deformable image registration of patients' brain scans from different time points. Another interesting application would be to deploy the model towards the discovery of novel prognostic markers. In particular, markers such as midline shift [9,98] or lateral ventricle displacement [99] are often used as indirect measures of mass effect and they show a strong correlation with survival. These markers, however, measure only a displacement of a single point. On the other hand, the model captures the full deformation field in a three-dimensional context. In this way, the displacement of multiple brain landmarks can be quantified and correlated with the patient survival/prognosis, potentially revealing new predictive markers.

A limitation of the proposed model is that it does not account for the circulation of CSF within and outside of the brain. As a consequence, the model cannot capture swelling of the ventricles, which can happen in some patients owing to the presence of the tumour or as a response to the treatment. Future works should thus account for the role of CSF circulation and its effect on ventricular deformations. Of particular interest would also be model extension accounting for distortion of the brainstem, caused either by CSF occlusion along the brainstem or by tumour infiltration into the brainstem itself, which have been recently identified as characteristic features presented in final-stage glioblastoma patients [100].

In the future the model can also be extended by incorporating various factors such as anisotropic tumour infiltration informed by patient-specific DTI [49,78,101], stress-constrained tumour invasion [79], the Allee effect [102] or go-or-grow principle [103,104]. Another compelling direction is to model gross tumour resection and consequent changes. The resection can be modelled by removing the tumour and the resection path (i.e. the tissue leading from the skull to the tumour) and by replacing the void with CSF. The resection cavity can be estimated from the postoperative scans. The presented model can then be used to simulate the progression and mass effect of the residual tumour in the absence of treatment, including tumour-induced compression of the resection cavity. However, the postoperative brain anatomy undergoes significant tumour-unrelated changes such as tissue decompression, which are not captured by the current model. The model only considers the tumour-induced pressure and mass effect. Once the tumour is resected, the pressure source is removed and the tumour-induced pressure drops to zero, i.e. to the patient's normal ICP. Although this is compatible with the outcome of surgery, the uniform pressure in the model does not generate the deformation force to drive the tissue decompression. This could be overcome by including a sink term for the pressure inside the resection cavity to drive the tissue relaxation. The relaxation of residual stresses can also drive tissue decompression (e.g. [10,105–109]). Further studies are however required to understand and correctly capture postoperative brain biomechanical changes.

To facilitate model calibration from patient data, the proposed model relies on a small number of parameters. The model calibration and accuracy of patient-specific predictions should be tested on a larger patient cohort in the following studies. The work presented here serves as a proof of concept that the proposed model holds the potential to capture glioma progression and accompanying tissue deformations and pressure dynamics in the patient's brain. These results encourage future studies of patient-specific model predictions aiming to assist clinical interventions by providing non-invasive estimates about the patient's state and the disease progression over time. Finally, to facilitate deployment of the proposed methodology, a highly parallel implementation of the model is publicly released.

## Data Availability

All code used in this work is publicly available in https://github.com/JanaLipkova as the following repositories: *GliomaSolver*—the model and tutorial;^1^ *Registration*—image registration;^2^ and *S3*—tissue segmentation tool.^3^

## Endnotes

^1^https://github.com/JanaLipkova/GliomaSolver.

^2^Tissue segmentation software: https://github.com/JanaLipkova/s3.

^3^Automated image-registration tool: https://github.com/JanaLipkova/Registration.
